# Supplementary material for: Efficient analysis of large datasets and sex bias with ADMIXTURE
Source: BMC Bioinformatics. 2016 May 23;17:218. doi: 10.1186/s12859-016-1082-x (PMC4877806; doi:10.1186/s12859-016-1082-x)
Supplement: Additional file 1 — Supplementary Text. (PDF 132 kb) [file 12859_2016_1082_MOESM1_ESM.pdf]

Supplementary Text for Efficient analysis of large datasets and sex bias with ADMIXTURE

Suyash S. Shringarpure, Carlos D. Bustamante,
Kenneth Lange, David H. Alexander

S1 Evaluating the accuracy of estimated allele frequencies

Another way of measuring the discrepancy between the estimated allele frequencies and the ExAC allele frequencies that takes into account the effect of frequency is to use the binomial deviance, defined for n SNPs as $D = \sum_{i=1}^{n} f^i_{true} \log\left(\frac{f^i_{true}}{f^i_{estimated}}\right) + (1-f^i_{true}) \log\left(\frac{1-f^i_{true}}{1-f^i_{estimated}}\right)$ where f^i_{true} and $f^i_{estimated}$ are the true (ExAC) and estimated allele frequencies for the ith SNP. We find that the binomial deviance for the allele frequency estimates using the unrelated individuals only (7.22) is less than the binomial deviance for the allele frequency estimates using all individuals (7.60), in agreement with our hypothesis that allele frequency estimates from the analysis using unrelated individuals are more accurate than those using all individuals.

S2 Behavior of the Wilcoxon signed-rank test under the null hypothesis

We examined the behavior of the Wilcoxon signed-rank test on data which we assume has no sex bias. We consider two such scenarios:

- **No sex bias between autosomes:** If there is no sex bias within autosomes, then comparing the ancestry on a single autosome to that on the rest of the autosomes should produce an empirical p-value distribution that is uniform under the null hypothesis.
- **No sex bias between two haplotypes in an autosome:** If there is no sex bias within autosomes, then comparing the ancestry on the two phased haplotypes should produce an empirical p-value distribution that is uniform under the null hypothesis.

S2.1 No sex bias between autosomes

For the ASW individuals from the 1000 Genomes Project used earlier, we compared ancestry on autosome N (N=15/16/17/18) to ancestry on the rest of the

autosomes using ADMIXTURE on the full set of 1087 individuals with K=3. Chromosomes 15, 16, 17 and 18 were chosen for this analysis since they have a comparable number of SNPs to chromosome X in the LD-thinned dataset.

We used the Wilcoxon signed-rank test to evaluate the statistical significance of the results. For each autosome, we obtained 3 p-values for the differences in ancestry, one for each ancestry component. Overall, we obtained 12 p-values for the 4 autosomes. For a well-behaved test, these p-values should follow a uniform distribution since they were generated under the null hypothesis (of no sex bias). A one-sample Kolmogorov-Smirnov test for goodness-of-fit fails to reject the hypothesis that these p-values were generated from a uniform distribution (p=0.11). Using only 2 of the 3 ancestry components to reduce correlation between p-values also produces similar results. Thus, the test is well-behaved.

S2.2 No sex bias between two haplotypes in an autosome

Assuming no sex bias in the autosomes, we expect no systematic differences in ancestry between the two haplotypes of a single autosome (or multiple autosomes). We tested this by extracting the two phased haplotypes for chromosomes 15,16,17 and 18 for the 1087 individuals from the 1000 Genomes project and running ADMIXTURE with K=3. We then compared the following ancestries for the ASW individuals pairwise for chromosome N (N=15/16/17/18):

- Ancestry on haplotype 1
- Ancestry on haplotype 2
- Ancestry on the entire chromosome N

Thus, for each ancestry component and chromosome, we generated 3 p-values from pairwise comparisons, for a total of 36 p-values (= 3×3 ancestry components × 4 chromosomes). A one-sample Kolmogorov-Smirnov test for goodness-of-fit fails to reject the hypothesis that these p-values were generated from a uniform distribution (p=0.19). Using only 2 of the 3 ancestry components and/or using only 2 of the 3 pairwise comparisons to reduce correlation between p-values also produces similar results. Thus, the test is well-behaved.
